# Social exclusion modulates fairness consideration in the ultimatum game: an ERP study

**DOI:** 10.3389/fnhum.2013.00505

**Published:** 2013-08-23

**Authors:** Chen Qu, Yuru Wang, Yunyun Huang

**Affiliations:** Psychology Research Center, School of Psychology, South China Normal UniversityGuangzhou, China

**Keywords:** social exclusion, fairness consideration, outcome evaluation, event-related potential (ERP), feedback-related negativity (FRN)

## Abstract

Previous neuroimaging research has identified brain regions activated when people’s fairness consideration changes under conditions of social exclusion. The current study used EEG data to examine the temporal process of changes in fairness consideration under social exclusion. In this study, a Cyberball game was administered to manipulate participants’ social exclusion or inclusion. Then, in the following Ultimatum game (UG), participants’ brain potentials were recorded while they received fair/unfair offers from someone who previously excluded them, someone who previously included them, or a stranger. Results showed that feedback-related negativity (FRN) after onset of distribution outcome was more pronounced for unfair offers compared to fair offers. Moreover, the FRN was more negative-going in response to unfair offers from people who previously excluded them than from the includer and the stranger. Fair offers elicited a larger P300 than unfair offers. In addition, P300 was more positive-going for unfair offers from the stranger than from the excluder and the includer. This study reveals a temporal process in which the effects of social exclusion on fair consideration are reflected in FRN in the early stage of outcome evaluation. These data also suggest that the FRN is modulated by the subjective evaluation of outcome events in a social context.

## Introduction

Human beings rely on group life for their health and well-being. Being accepted into a social group is therefore an important goal of human striving, and being excluded can be highly distressing. Previous researches have shown that social exclusion leads to stress, sadness, or anger, and that it threatens fundamental needs such as self-esteem, belonging and meaningful existence (Williams, 2001, 2007). Functional magnetic resonance (fMRI) studies have shown that social exclusion elicits activity in brain regions involved in affective processing and physical pain (Eisenberger et al., 2003; Eisenberger and Lieberman, 2004).

Given the strong human need for social belonging, social exclusion is likely to influence subsequent coping. As shown in previous studies, ostracized individuals have been shown to attend to and remember social information more than included individuals do (Gardner et al., 2000; Lakin et al., 2008). They are also more sensitive to social/emotional inconsistencies (Pickett et al., 2004; Bernstein et al., 2008). Furthermore, there is evidence that people’s behavioral responses are modulated by prior interactions; people are more willing to cooperate with those who included them before and they act less prosocially to people who excluded them (Maner et al., 2007; Hillebrandt et al., 2011).

Recently, Gunther Moor et al. (2012) investigated the neural networks that are sensitive to social exclusion and participants’ punishment behavior after exclusion. In their study, participants were asked to play a dictator game (DG; Kahneman et al., 1986) in which the allocator decided how to distribute assets and the recipient had no choice but to accept the allocation. Results showed that allocators selectively punished the excluders by making lower offers. In addition, providing offers to excluders activated the temporoparietal junction (TPJ), superior temporal sulcus (STS) and the lateral PFC. The TPJ and the STS are involved in perspective-taking and theory of mind (e.g., Gallagher and Frith, 2003; Pelphrey et al., 2003; Kross et al., 2007), and the ventrolateral prefrontal cortex (vlPFC) in the regulation of negative affect (Rilling and Sanfey, 2011). The authors suggested that these processes may be involved in the decision to punish the excluders and to maximize one’s own gains. It should be noted, however, that the study assessed the effects of social exclusion exclusively in terms of the allocator’s decision-making behavior. It is not clear how the recipient’s fairness consideration would be affected by social exclusion, and closer to the purpose of the present study, whether and how social exclusion would affect recipients’ fairness consideration in the early stage of outcome evaluation.

This study was therefore conducted to investigate whether the feedback-related negativity (FRN) effect associated with unfair offers is modulated by social exclusion. We used the event-related potential (ERP) technique to study the temporal process of fairness consideration under social exclusion. We employed Cyberball (Williams et al., 2000), an ostensibly “online” ball-tossing game, to manipulate participants’ feelings of being included or excluded. Participants subsequently played the ultimatum game (UG) in the role of recipient, experiencing fair and unfair outcomes. The UG was originally developed by Güth et al. (1982), and it is similar to the DG but has one difference: the recipient can either accept or reject the allocator’s offer. If accepted, the pie is divided as proposed; if rejected, both of them end up empty handed.

We focused on two ERP components that have been shown to be particularly sensitive to outcome evaluation and performance monitoring. The first component, FRN which has its source in anterior cingulate cortex (ACC), is a negative deflection peaking between 200 ms and 350 ms at frontocentral recording sites (Miltner et al., 1997; Holroyd and Coles, 2002; Hajcak et al., 2005, 2007). The FRN has been shown to be more pronounced for negative feedback associated with unfavorable outcomes, such as incorrect responses or monetary loss, than for positive feedback (Gehring and Willoughby, 2002; Yeung and Sanfey, 2004; Holroyd et al., 2006; Goyer et al., 2008). Further studies showed that these differential responses to decision outcome can be modulated by social factors, such as the extent of personal responsibility for the outcome (Li et al., 2010) and an interpersonal relationship between the evaluator and the decision maker (Fukushima and Hiraki, 2006, 2009; Itagaki and Katayama, 2008). These studies suggest that the FRN reflects the motivational/affective evaluation of the outcome events: the FRN may reflect the subjective importance of the outcome for an individual (Gehring and Willoughby, 2002; Pailing and Segalowitz, 2004; Boksem et al., 2011). Importantly, recent studies found that violations of social norms that create negative outcomes in the social domain, such as unfair or unequal offers in asset division, also elicit more negative-going FRN than fair offers (Boksem and De Cremer, 2010; Hewig et al., 2011; Wu et al., 2011).

Based on these studies, we predicted a larger FRN for the unfair offers, as compared to the fair offers received by participants in an UG. Importantly, this FRN effect was predicted to be modulated by social exclusion. As a recipient in UG, participants received unfair offers from people who had excluded them, people who had included them, and from a stranger. Recipients may become more sensitive to the excluder than others, particularly in a social interaction context, resulting in more negative-going FRN in unfair offers from the excluder than from others. We also examined whether another ERP component, P300, would be modulated by the experimentally induced social exclusion. The P300 shows the most positive peak in the period of 200–600 ms post-onset of feedback and it typically increases in magnitude from frontal to parietal electrodes; if this effect is modulated by social exclusion, we would see changes in P300 after social exclusion but not after kinds of interaction.

## Methods

### Participants

Twenty undergraduate and graduate students (10 females) were recruited from the University intranet. After the experiment, two participants stated that they disbelieved the setup of the experiment, and two participants displayed excessive artifacts in the EEG recording. These participants were excluded from data analysis. The mean age of the participants was 22.1 years, ranging between 19 and 25 years. They were paid 20 Chinese Yuan (about $3) as basic payment and were informed that additional monetary rewards would be paid according to allocators’ offers and their decisions in the task, although in the end all participants were paid 35 Yuan (about $5) extra on top of the basic payment.

All participants were right-handed and had normal or corrected-to-normal vision. They had no history of neurological or psychiatric disorders. Informed consent was obtained from each participant before the test. This study was approved by the Ethics Committee of the State Key Laboratory of Cognitive Neuroscience and Learning at Beijing Normal University.

### Design and procedures

The design was a 2 × 3 within-subjects factorial with the first factor referring to the level of fairness (Fair, Unfair) and the second factor referring to the condition (Inclusion, Exclusion and Strangeness). Fair offers could be 40 or 50 Yuan (out of 100 Yuan) whereas unfair offers could be 20 or 10 Yuan.

Participants performed two tasks. Firstly, participants played Cyberball, which started with an inclusion game, followed by an exclusion game to induce feelings of social inclusion and exclusion, respectively. After Cyberball, participants acted as recipients in an UG.

#### Cyberball

Before playing Cyberball, participants were presented with an instruction screen showing that they would play an online ball-tossing game with two other players whose photographs they would see throughout the game. In reality participants played against algorithms; in order to create a realistic atmosphere, the game started with a loading screen notifying that the computer was trying to connect to the other players. The photographs of the fictitious players were taken from the Chinese Affective Picture System. During the experiment, participants were led to believe that this was a study about the relation between task performance and mental visualization and that they had to try to imagine the game as vividly as they could (van Beest and Williams, 2006).

**Figure 1:**
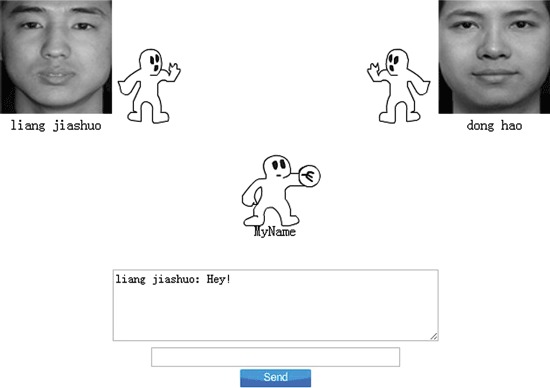
**Screenshot of a Cyberball game interface.** Participants are represented by a cartoon hand at the bottom of the screen, and the other two (fictional) players’ photographs are shown in the upper corners of the screen.

During Cyberball, participants played an animated ball-tossing game. The photographs of the other (fictitious) players were displayed as animated cartoon hands at the top of the screen (Figure 1). In order to control for gender effects, everyone believed they were playing with same-gender players. There were 30 throws of the ball in the inclusion condition, and 30 in the exclusion condition. In the inclusion condition, participants were thrown the ball approximately half the time by each of the other two players. Once receiving the ball, participants could throw the ball back to one of the players by clicking the mouse. Participants were then told that they would play a similar game with two novel players, which in fact were two new fictitious partners. This next game was the exclusion game. After two initial throws, participants were not thrown the ball again.

#### Ultimatum game

After the Cyberball games, the participants entered an UG during which EEG data was collected. The two tasks (games) run in the same room. And the electrodes were attached before Cyberball game. They were informed of the rules of UG, that they would play as a recipient, and that the allocator would be one of the players with whom they had played Cyberball or a novel player (stranger).

Participants were seated in a quiet room approximately 150 cm from a computer screen with the horizontal and visual angles below 5°. To become acquainted with the task, the participants completed six practice trials.

**Figure 2:**
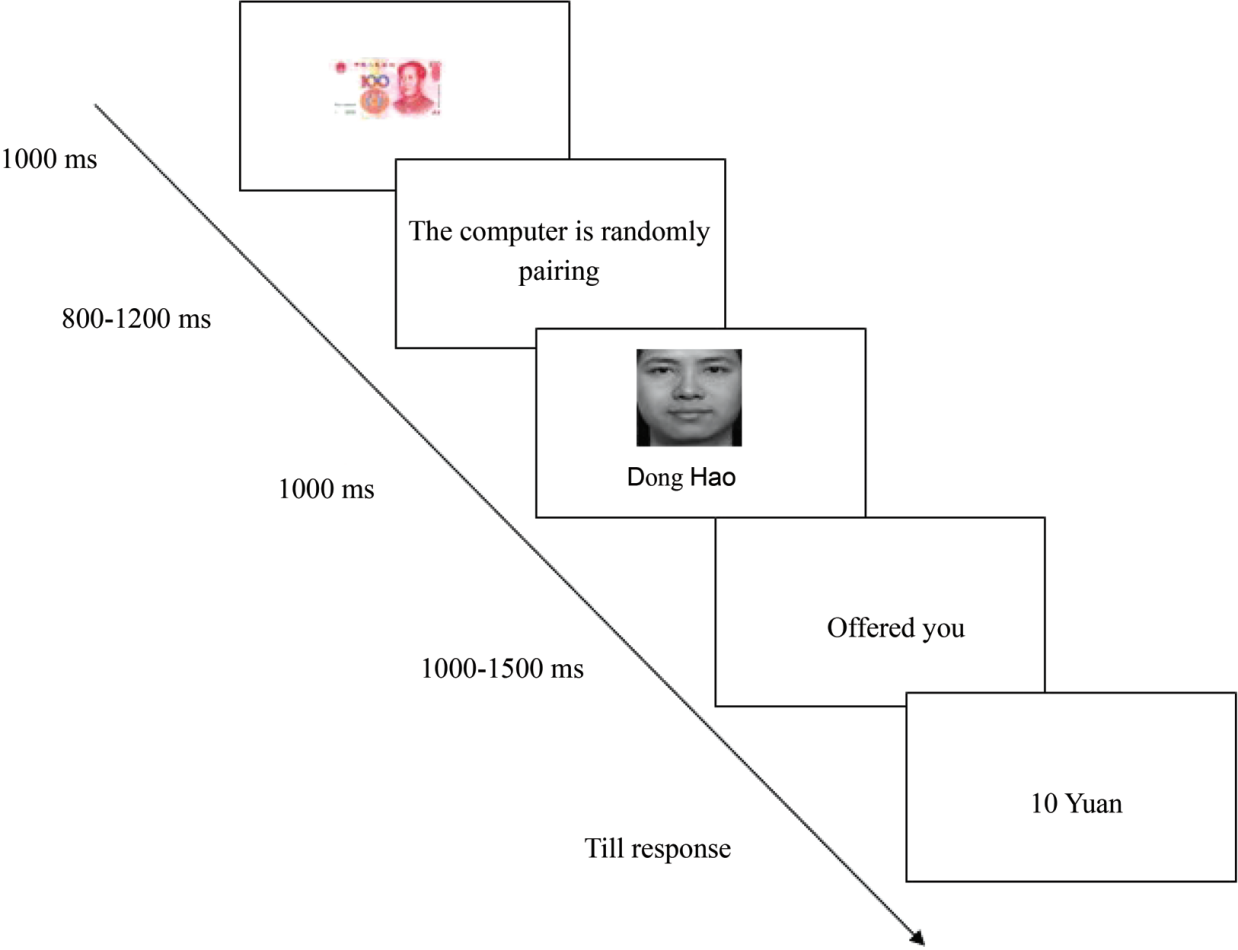
**Sequence of events in a single trial in the ultimatum game**.

The time course of a trial is illustrated in Figure 2. Each trial was initiated with the presentation of a 100 Yuan (about $15) bill for 1000 ms duration. Subsequently, the sentence “The computer is randomly pairing” in Chinese was presented (800~1200 ms), suggesting that the other person was randomly selected to play as an allocator in the current round of the game. Then the allocator’s head portrait was presented at the center of the screen for 1000 ms. After the word “offered you” in Chinese was presented for 1000~1500 ms the amount was shown to the recipient, and the participants were asked to choose “accept” or “reject” by pressing the “F” or “J” key on the keyboard. The next trial began 1 second after the offset of the feedback.

The experiment consisted of 5 blocks of 78 trials each, and each of the six experimental conditions had 60 trials. In addition, another 30 trials with an offer of 30 Yuan (out of 100 Yuan) were used as fillers, in order to increase credibility. Without the participants’ knowledge, all the offers were predetermined by a computer program. The participants were debriefed, paid and thanked at the end of the experiment.

### Manipulation check

After each Cyberball game, participants completed a post-experimental four-item questionnaire to assess their current mood. Using a scale from 1 to 9, they rated their mood as feeling good/bad, happy/sad, relaxed/tense, and friendly/unfriendly (Aron et al., 1992; Sebastian et al., 2010). Lower scores indicate lower mood.

### EEG recording and analysis

EEGs were recorded from 32 scalp sites using tin electrodes mounted in an elastic cap (NeuroScan Inc., USA) according to the international 10–20 system. The vertical electrooculograms (VEOGs) were monitored with electrodes located in four places: above and below the right eye and 1.5 cm lateral to the left and right external canthi. All EEGs and EOGs were referenced online to the left mastoid and off-line algebraic re-referenced to the average of the left and right mastoids. All electrode impedances were kept below 5 kΩ for all recordings.

Off-line analysis was performed using Brain Vision Analyzer software (Brain Products). The electrophysiological signals were amplified with a band pass of 0.01–100 Hz and continuously digitized at a rate of 500 Hz. Ocular artifacts were corrected with an eye-movement correction algorithm that employs a regression analysis in combination with artifact averaging (Semlitsch et al., 1986). Trials in which EEG voltages exceeded a threshold of ± 80 μV during the recording epoch were excluded from further analysis. EEG data were digitally filtered below 30 Hz (24 dB/Octave) for all recordings. EEG epochs of 1000 ms (with a 200-ms pre-stimulus baseline) were extracted offline for ERPs time-locked to the onset of offers from the allocators.

Because previous studies (Gehring and Willoughby, 2002; Hajcak et al., 2005, 2007) have shown that the FRN is maximal on midline frontocentral electrodes, a pooling of the Fz, FCz, and Cz electrodes was analyzed. The P300 is found to be maximal on posterior sites (Yeung and Sanfey, 2004) and analyses were therefore restricted to a pooling of the CPz and Pz electrodes. And we presented ERPs data on each electrode (submitted for statistical analysis) in Tables 1, 2.

**Table 1:** **The mean amplitudes (μV) in FRN for each condition**.

	**Inclusion *M*(*SD*)**		**Exclusion *M*(*SD*)**		**Novelty *M*(*SD*)**	
	**fair**	**unfair**	**fair**	**unfair**	**fair**	**unfair**
Fz	2.833 (5.169)	.110 (5.347)	2.244 (5.554)	−.680 (5.553)	1.674 (5.746)	.167 (5.135)
FCz	3.459 (5.054)	1.007 (5.201)	3.145 (5.136)	.379 (5.217)	2.636 (5.588)	1.183 (5.117)
Cz	5.253 (4.586)	3.197 (4.466)	4.991 (4.733)	2.347 (4.208)	4.662 (4.701)	3.455 (4.537)

Notes: M, arithmetic mean; SD, standard deviation

**Table 2:** **The mean amplitudes (μV) in P300 for each condition**.

	**Inclusion *M*(*SD*)**		**Exclusion *M*(*SD*)**		**Novelty *M*(*SD*)**	
	**fair**	**unfair**	**fair**	**unfair**	**fair**	**unfair**
CPz	8.145 (4.328)	5.323(3.928)	7.632(4.165)	5.470(3.759)	7.759(4.216)	6.618 (3.875)
Pz	9.211 (4.119)	6.427(3.821)	9.100 (4.196)	6.734 (4.047)	8.977 (4.128)	7.786 (4.006)

**Figure 3:**
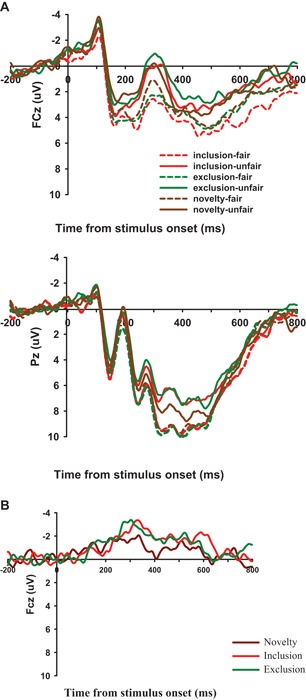
**(A)** ERP responses time-locked to the onset of different offers at the midline FCz, and Pz. The 240–340 ms time window was for the calculation of the mean amplitudes of the FRN. **(B)** Unfair minus fair difference wave in Inclusion, Exclusion, and Novelty condition at FCz.

Average amplitudes over these electrodes in each region were used in the following analyses, using classical definitions concerning the time windows of the FRN and the P300 (Gehring and Willoughby, 2002; Yeung and Sanfey, 2004) and according to visual inspection of grand-averaged waveforms (Figure 3A) in the present experiment. The ERP components analyzed included FRN (the mean amplitudes in the time window of 240–340 ms) and P300 (the mean amplitudes in the time window of 320–420 ms). Separate repeated measures analyses of variance (ANOVAs) were conducted for the two potentials with three within-participant factors: fairness level (Fair, Unfair), condition (Inclusion, Exclusion and Strangeness) and electrode locations (FRN: Fz, FCz, and Cz; P300: CPz and Pz). We also compared the average amplitudes of the FRN (260–300 ms) difference waves for each condition (Figure 3B). Following the prior studies (Holroyd and Krigolson, 2007; Chen et al., 2012), we calculated the FRN by subtracting the Fair-FRN from the Unfair-FRN. A two-way ANOVA was conducted on the difference waves. ANOVA factors were condition (Inclusion, Exclusion and Strangeness/Novelty) and electrode site (Fz, FCz, and Cz). The Greenhouse-Geisser correction for violation of the assumption of sphericity was applied where appropriate. The Bonferroni correction was used for multiple comparisons. Furthermore, Pearson correlations (two-tailed) were run to evaluate putative links between the emotionality and FRN amplitude.

## Results

### Manipulation checks of social exclusion

Participants reported lower mood ratings for all mood constructs (good/bad, happy/sad, relaxed/tense, and friendly/unfriendly) after the exclusion game than after the inclusion game (results of all paired sample t-tests significant at *p* < 0.010). When the four mood ratings were averaged to create an overall index of mood, the results of the paired-sample t-test showed over all lower mood after the exclusion game than after the inclusion game, *t*(15) = 4.841, *p* < 0.001.

### Behavioral performance

ANOVA using the rejection rate as the dependent measure and fairness level (Fair, Unfair) and condition (Inclusion, Exclusion and Strangeness) as two within-subject variables, revealed a significant main effect of fairness level, *F*(1,16) = 222.460, *p* < 0.001, indicating that the rejection rate for unfair offers (0.889 ± 0.052) was higher than for that for fair offers (0.036 ± 0.029). The main effect of condition (allocator type) and the interaction between fairness level and condition were not significant.

### ERP responses to the presentation of division schemes

#### FRN

For the FRN amplitude, a 2 (fairness level: Fair, Unfair) × 3 (condition: Inclusion, Exclusion and Novelty) × 3 (electrode location: Fz, FCz, Cz) repeated-measures ANOVA showed a significant main effect of electrode location, *F*(2, 30) = 17.535, *p* < .001. Post-hoc analyses showed a more negative FRN at Fz (1.058 μV ± 1.330) than FCz (1.968 μV ± 1.271) and Cz (3.984 μV ± 1.094), *p* < .05. The main effect of fairness level was also significant, *F*(1,15) = 44.119, *p* < 0.001, indicating that FRN was more negative-going for unfair offers (1.241 ± 1.197 μV) than for fair offers (3.433 ± 1.227 μV). Importantly, the interaction between fairness level and condition (allocator type) was significant, *F*(2, 30) = 12.430, *p* = 0.006, with FRN more negative-going for unfair offers from the excluder (0.682 ± 1.210 μV) than for offers from the stranger (1.602 ± 1.205 μV) and the includer (1.438 ± 1.219). The differences were all significant, *p* = 0.030 and *p* = 0.009.

The ANOVA performed on the difference waves of FRN amplitude showed that the main effect of condition was significant [*F*(2,30) = 11.954, *p* < 0.001], with a larger FRN in the exclusion condition (−3.084 ± 0.307 μV) than in the novelty condition (−1.167 ± 0.343 μV) and the inclusion condition (−2.196 ± 0.480 μV) [*p* < 0.001, *p* = 0.049]. In addition, the main effect of electrode was also significant [*F*(2, 30) = 4.569, *p* = 0.019], with more negative FRN at Fz and FCz than at Cz.

#### Correlations with mood change

A Pearson correlation indicated that bigger change of mood (the amount of negativity of mood was given by subtracting the ratings on exclusion game from the ratings on inclusion game) was related to more negative FRN during the unfair condition, *r* = −0.529, *p* = 0.043. We further computed the correlations for the three different kinds of allocators. The negative correlation between the mood change and the FRN for unfair offers from the excluder or the stranger, was significant (*r* = −0.540, *p* = 0.038; *r* = −0.555, *p* = 0.032); while the correlation for the includer condition was not significant, *r* = −0.473, *p* > 0.5. No significant correlation was found for mood change and the FRN during the fair condition, *P* > 0.9.

#### P300

For the mean amplitudes in the 320–420 ms (P300) time window, the 2 × 3 × 2 ANOVA also showed a significant main effect of electrode location, *F*(1, 15) = 10.935, *p* = 0.005, with a more positive P300 at Pz (8.370 ± 1.063) than at CPz (7.086 ± 1.018). The main effect of fairness level was significant, *F*(1, 15) = 26.715, *p* < 0.001, indicating that the mean amplitudes were more positive for fair offers (8.841 ± 1.080 μV) than for unfair offers (6.615 ± 1.008 μV). The interaction between fairness level and condition (allocator type) reached significance as well, *F*(2, 30) = 6.846, *p* = 0.004, indicating that the P300 for unfair offers from the stranger (7.464 ± 1.042 μV) was more positive-going than for unfair offers from the excluder (6.092 ± 1.053 μV), *p* = 0.036. The P300 for unfair offers from the stranger was larger than for unfair offers from the includer (6.290 ± 1.036 μV) but the difference didn’t reach the significance.

## Discussion

Prior literature (Gunther Moor et al., 2012) on fair consideration under social exclusion is difficult to the temporal course of allocators’ evaluation on fair and unfair offer. Using ERP technique, this study employed UG game to explore the recipients’ evaluation process. Results showed that FRN, an early component related to outcome evaluation, was modulated by social exclusion—previous encounters with people who had included or excluded them may affect people’s consideration of fairness. More specifically, the FRN was more negative-going in response to unfair offers from people who previously excluded them than from the includer and the stranger, which adds new evidence that social exclusion affects fairness consideration in the early stage of outcome evaluation.

Self-report ratings showed that all participants reported greater distress after exclusion, which set the stage for examining subsequent fairness considerations. The use of both behavioral and electrophysiological data provided a rich set of information about responses to social exclusion. Behaviorally, participants were more reluctant to accept unfair offers than fair offers, regardless of their past interactions with the person making the offer. A similar pattern emerged in the electrophysiological data. FRN has been shown to reflect the neural activity that is associated with negative events such as error commission, receiving negative feedback and incurring financial losses (Gehring and Willoughby, 2002; Yeung and Sanfey, 2004; Holroyd et al., 2006; Goyer et al., 2008), and as such is a particularly helpful measure of humans’ response to social exclusion. In the current study, unfair offers (which are against social norms and reflect negative outcomes in the social domain) elicited more negative going ERP responses compared to fair offers in FRN time window (240–340 ms). However, this FRN was also affected by allocator condition: the FRN was more negative when the unfair offers were from the person who excluded the participant, compared with unfair offers from the includer and the stranger. Moreover, the P300 was more positive for fair offers than for unfair offers, and more positive for unfair offers from the stranger than from the excluder and the includer.

Importantly, the results revealed that unfair offers from the excluder elicited a larger FRN than did unfair offers from the includer and stranger. It has been proposed that FRN amplitudes are most dependent on how concerned subjects are over decision outcome, especially in a social context, in other words, FRN reflects subjective outcome value (Leng and Zhou, 2010; Boksem et al., 2011; Luo et al., 2011; Wu et al., 2011; Long et al., 2012). Indeed, the researchers have found that being out performed by peers or being treated unfairly especially for the people who were more concerned about the social rule, results in a more negative-going FRN. Thus, FRN amplitude may reflect how engaged participants are in processing cues that indicate possible threat to their standing in the social group. In humans, as social animals, the neural system involved in the detection of errors or losses and performance evaluation (as reflected by the FRN), may be particularly involved in processing social punishment or threat of which social exclusion can be a very prominent one. Indeed, also the ACC activity, which is the putative source of the FRN, has been associated with punishments and losses in the social domain, such as being rejected or being treated unfairly (Gehring and Willoughby, 2002; Eisenberger et al., 2003; Boksem and De Cremer, 2010; Zhou and Wu, 2011).

Particularly, in the current study, participants who experienced exclusion in the Cyberball game were subsequently more sensitive to the excluder’s allocation in the UG. Given an unfair split from the excluder, participants as recipients might feel hurt a second time. More specifically, the unfair split from the excluder is perceived by the participants as more socially negative than that from the includer and stranger, which was reflected in an enhanced FRN elicited by unfair offers from the excluder. This suggests that participants integrated social information in the early stage of outcome evaluation.

The significant correlation between the mood change and the FRN amplitude indicated that increasingly levels of negative emotionality were associated with more negative FRN induced by unfair offers, and the finding is consistent with previous studies (Mak et al., 2009; Santesso et al., 2012). Additionally, the difference among three conditions (the correlation varied according to the allocator condition) showed that purely cognitive model of outcome evaluation may not adequately explain the variation in response to feedback, and the context or state should be considered in FRN research.

The result of a main effect of fairness level on the P300, with more positive responses to fair offers than to unfair offers, is consistent with prior researches on the functional significance of P300 in outcome evaluation. Previous studies have indicated that the P300 is related to processes of attentional allocation and/or high-level motivational/affective evaluation (Donchin and Coles, 1988; Nieuwenhuis et al., 2004, 2005; Yeung and Sanfey, 2004; Sato et al., 2005). According to equity theory (Peters et al., 2008), individuals who are facing inequity would feel less satisfied with asset distribution than would individuals who are facing equity. The stronger P300 responses to fair offers than to unfair offers might suggest that participants (recipients of asset distribution) in this study attached more motivational/affective significance to the fair divisions than to unfair divisions.

In addition, the P300 data showed an interaction between allocator condition and fairness level: unfair offers from the stranger elicited more positive P300 than unfair offers from the excluder and the includer. Although both types of offers (unfair offers from people who played ball games or a total stranger) violate the equity rule of social norms, it is possible that different amounts of attentional resources are used to process the two types of allocators. Participants might be less familiar with the stranger than with the people who played Cyberball with them before. However, as the difference between the novelty-unfair offer and the inclusion-unfair offer was not significant, we should be careful about this inference.

As for the behavioral results, the high acceptance rate to all fair offers in all three groups (that is, the absence of an initial social exclusion effect) may result from individuals not only caring about fairness in asset distribution, but also being self-interested. Self-interest is mostly likely to be shown when it is unlikely to be negated (Rabin, 1993; Kagel and Wolfe, 2001). In this situation, effects of other social factors (including exclusion) are dwarfed or over-shadowed.

The present study might have limitations in that that the social context was manipulated experimentally. In future research it would be of interest to test whether relationships in real life similarly modulate fairness considerations. Most people, in real life, experience periods of social exclusion, and exclusion hurts (MacDonald and Leary, 2005). And people have to deal the relationship with those who (a stranger or familiar people) exclude them well, which is important for our mind health. As hard as it may seem when we are experiencing anger towards someone, the key to overcoming the emotion lies first in understanding and finally in working out. In addition, in this experiment, the manipulation of fairness level was polarized—fair or unfair. It is not clear how people react to relatively less unfair offers (e.g., 30% of the total, rather than 0%), or under which circumstances people may reject the excluder more than others (Twenge et al., 2001; Buckley et al., 2004; Maner et al., 2007).

In summary, the present study reveals evidence that social exclusion affects fair consideration. Results showed that FRN was more negative-going for unfair offers than for fair offers and the differential effect was modulated by social exclusion, with the FRN being more negative-going to unfair offers from the excluder than to similar offers from the stranger and the includer. Similarly, the P300 in the central-posterior region was more positive for fair than for unfair offers, and was also affected by social context. Therefore we suggest that social exclusion could affect recipients’ fairness consideration in the early stage of outcome evaluation. Moreover, the FRN may reflect the subjective evaluation of outcome events in a social context rather than function as a general mechanism that evaluates whether the offer is consistent or inconsistent with the equity rule.

## Conflict of interest statement

The authors declare that the research was conducted in the absence of any commercial or financial relationships that could be construed as a potential conflict of interest.
